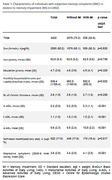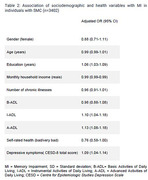# Objective memory impairment in individuals with memory complaints: baselinedata from the Brazilian Longitudinal Study of Aging (ELSI‐Brazil)

**DOI:** 10.1002/alz.093047

**Published:** 2025-01-03

**Authors:** Pedro JDMR Pinho, Laiss Bertola, Lucas Martins Teixeir, Matheus Ghossain Barbosa, Maria Fernanda Lima‐Costa, Cleusa P Ferri

**Affiliations:** ^1^ Universidade Federal de São Paulo (UNIFESP), São Paulo, São Paulo/SP Brazil; ^2^ Hospital Alemão Oswaldo Cruz, São Paulo, São Paulo Brazil; ^3^ Fundação Oswaldo Cruz and Universidade Federal de Minas Gerais – Núcleo de Estudos em Saúde Pública e Envelhecimento, Belo Horizonte Brazil

## Abstract

**Background:**

Subjective Memory Complaints (SMC) are common with aging and might be associated with objective memory impairment (MI) and with higher risk to develop dementia. This association is understudied in socially and ethnically diverse populations, such as Brazilians.

**Method:**

This study uses the baseline data from the ELSI‐Brazil, a representative sample of 9402 Brazilians over 50 years. We used the question “Currently, how do you rate your memory?” to classify the participants as without SMC (excellent/very good/good) and with SMC (fair/bad). Memory impairment (MI) was defined through a z‐score extracted from a regression based‐norms approach (based on their age, education, and gender/sex) derived from a healthy subset of individuals from the ELSI‐Brazil study. The memory score is based on a combination of immediate and delayed response from the episodic memory test (10‐word list). Other data about sociodemographic factors (sex, age, education, income), depressive symptoms, self‐rated health, number of chronic illnesses, and activities of daily living: basic (BADL), instrumental (IADL) and advanced (AADL) were measured. We used logistic regression with robust variance to estimate the association between the above variables and MI.

**Result:**

From the original sample, 7831 participants had no missing data for all variables, with 3402 (41.9%) individuals with SMC and included in this study. People with SMC had a mean age of 62.6 years (SD = 9.3), a mean education of 4.7 years (SD = 3.9), and 24.8% had MI (table 1). Logistic regression shows that, in individuals with SMC, higher education, greater impairment in IADLs and AADLs, and a higher number of depressive symptoms were associated with MI. However, higher household income per capita were associated with less MI (table 2).

**Conclusion:**

While SMC affects 42% of the original sample, objective MI affects only a quarter of these individuals, showing that most of them have complaints but not objective memory deficits. Memory impairment in individuals with memory complain is associated with higher education, lower income, depressive symptoms, and disability in instrumental and advanced activities of daily living.